# MTA1 drives malignant progression and bone metastasis in prostate cancer

**DOI:** 10.1002/1878-0261.12360

**Published:** 2018-08-14

**Authors:** Avinash Kumar, Swati Dhar, Gisella Campanelli, Nasir A. Butt, Jason M. Schallheim, Christian R. Gomez, Anait S. Levenson

**Affiliations:** ^1^ Arnold & Marie Schwartz College of Pharmacy and Health Sciences Long Island University Brooklyn NY USA; ^2^ Cancer Institute University of Mississippi Medical Center Jackson MS USA; ^3^ Department of Pathology University of Mississippi Medical Center Jackson MS USA; ^4^ Department of Radiation Oncology University of Mississippi Medical Center Jackson MS USA; ^5^ Present address: Department of Pediatrics Feinberg School of Medicine Northwestern University Chicago IL USA; ^6^ Present address: Anne Arundel Medical Center Annapolis MD USA; ^7^ Present address: Veterinary Medicine Long Island University Brookville NY USA

**Keywords:** bone metastasis, cathepsin B, intracardiac xenografts, luciferase imaging, MTA1, prostate cancer

## Abstract

Prostate cancer often metastasizes to the bone, leading to morbidity and mortality. While metastasis‐associated protein 1 (MTA1) is highly overexpressed in metastatic tumors and bone metastatic lesions, its exact role in the development of metastasis is unknown. Here, we report the role of MTA1 in prostate cancer progression and bone metastasis *in vitro* and *in vivo*. We found that MTA1 silencing diminished formation of bone metastases and impaired tumor growth in intracardiac and subcutaneous prostate cancer xenografts, respectively. This was attributed to reduced colony formation, invasion, and migration capabilities of MTA1 knockdown cells. Mechanistic studies revealed that MTA1 silencing led to a significant decrease in the expression of cathepsin B (CTSB), a cysteine protease critical for bone metastasis, with an expected increase in the levels of E‐cadherin in both cells and xenograft tumors. Moreover, meta‐analysis of clinical samples indicated a positive correlation between MTA1 and CTSB. Together, these results demonstrate the critical role of MTA1 as an upstream regulator of CTSB‐mediated events associated with cell invasiveness and raise the possibility that targeting MTA1/CTSB signaling in the tumor may prevent the development of bone metastasis in prostate cancer.

**Abbreviations**
ChIP‐Seq: chromatin immunoprecipitation sequencing
CTSB: cathepsin B
E‐cad: E‐cadherin
EMT: epithelial‐to‐mesenchymal transition
IHC: immunohistochemistry
MMP: matrix metalloproteinase (enzyme)
MTA1: metastasis‐associated protein 1
MUC1: mucin 1
NS: nonsilencing
NuRD: nucleosome remodeling and deacetylation (complex)
PI3K: phosphoinositide 3‐kinase
PTEN: phosphatase and tensin homolog deleted in chromosome 10
R26: ROSA26 locus on mouse genome
RPMI 1640: Roswell Park Memorial Institute (medium)
s.c.: subcutaneous
SAHA: suberoylanilide hydroxamic acid

## 1 Introduction

Bone metastases occur in about 80% of patients with advanced castrate‐resistant prostate cancer. As a major cause of morbidity and mortality in patients with prostate cancer, bone metastases are of great clinical concern. The mechanisms of trafficking and homing of aggressive prostate cancer cells into the bone have been previously investigated (Futakuchi *et al*., 2016; Sakamoto and Ichikawa, 2014; Ziaee *et al*., 2015). However, the exact molecular mechanisms involved remain elusive, which restricts the development of new and targeted therapeutics. Identification of bone metastasis driver molecules in the primary prostate tumors might help to target them at early stages of the disease, which can provide a shift of the disease progression toward indolent rather than the aggressive disease.

In our previous studies, we have identified metastasis‐associated protein 1 (MTA1) as a novel factor involved in ‘vicious cycle’ of prostate cancer cells interacting with bone cells, and hypothesized that MTA1 may play a specific role in prostate cancer bone metastasis (Kai *et al*., 2011). Our following studies validated high MTA1 expression in advanced metastatic prostate cancer and bone lesions of patients with prostate cancer (Dias *et al*., 2013a; Kai *et al*., 2011).

MTA1 belongs to the MTA family of proteins that are critical participants of the nucleosome remodeling and deacetylation (NuRD) multiprotein complex, which is involved in chromatin remodeling and gene silencing (Toh *et al*., 1994; Xue *et al*., 1998). Overexpression of MTA1 has been reported in various cancers (Levenson *et al*., 2014). We and others have demonstrated the correlation of MTA1 expression with clinicopathological measures of prostate cancer progression: high Gleason score, development of castrate‐resistant disease, recurrence, and bone metastasis (Dias *et al*., 2013a; Hofer *et al*., 2006; Kai *et al*., 2011). We have extensively reported on the critical role of MTA1 and its signaling at all stages of prostate cancer development and progression (Levenson *et al*., 2014). In addition to our initial demonstration of MTA1's functional role *in vitro* and in xenografts (Dhar *et al*., 2015a; Kai *et al*., 2010, 2011; Li *et al*., 2013), we recently demonstrated that MTA1 overexpression promotes molecular changes associated with inflammation‐mediated cancer initiation, epithelial‐to‐mesenchymal transition (EMT), and cancer progression linked to cell metastatic potential and angiogenesis in a multistage transgenic model of prostate cancer that closely mimics human disease (Butt *et al*., 2017; Dhar *et al*., 2016, 2017). Altogether, MTA1 is increasingly being recognized as an important upstream multifunctional regulator that affects various cellular responses, including cell adhesion, migration, and invasion in prostate cancer. However, the exact role of MTA1 and its downstream signaling mechanisms that mediate MTA1‐activated prostate tumor cell invasion and colonization into the bone are not well understood.

In this study, we systematically analyzed the *in vitro* and *in vivo* role of MTA1 in prostate tumor cell metastatic potential, tumor growth, and formation of metastasis using subcutaneous (s.c.) and experimental bone metastasis xenograft mouse models. We showed that MTA1 ablation profoundly suppressed the growth of prostate tumors and the ability of tumor cells to colonize the bone by inhibiting their migratory and invasive properties through repression of cathepsin B (CTSB), a cysteine protease that plays a critical role in bone metastasis (Podgorski *et al*., 2005; Withana *et al*., 2012).

## 2 Materials and methods

### 2.1 Cell culture

Prostate cancer cells, PC3M and LNCaP, were maintained in RPMI 1640 media containing 10% FBS as described previously (Butt *et al*., 2017; Dhar *et al*., 2016; Dias *et al*., 2013a,b; Li *et al*., 2013). PC3M cells expressing luciferase (PC3M‐Luc) were established, expanded, and tested for their luciferase activity using an IVIS Spectrum (PerkinElmer, Waltham, MA, USA). Cells were maintained in an incubator at 37 °C with 5% CO_2_. Cells were authenticated using short tandem repeat profiling at Research Technology Support Facility, Michigan State University.

### 2.2 MTA1 knockdown in prostate cancer cells

PC3M‐Luc cells were transduced with the three GIPZ MTA1 lentiviral shRNA, GIPZ GAPDH lentiviral shRNA, and GIPZ nonsilencing lentiviral shRNA. The GIPZ lentiviral shRNA system (GE Healthcare Dharmacon, Lafayette, CO, USA) contains the puromycin‐resistant gene for selection of transduced cells and TurboGFP for monitoring the selection under the fluorescence microscope. For the preparation of the viral particles, we used the pCMV‐ΔR8.91 packaging plasmid and the pMD.G envelope plasmid (Addgene, Cambridge, MA, USA). Cells were transduced using RPMI 1640 medium, which contained 4 μg·mL^−1^ polybrene (Sigma‐Aldrich, St. Louis, MO, USA) with lentiviral particles at a multiplicity of infection = 8. On day 2 post‐transduction, selection was initiated with 200 μg·mL^−1^ puromycin (Sigma‐Aldrich) and GFP‐positive clones were selected and propagated.

### 2.3 Western blot

Western blot analysis was carried out as described previously (Butt *et al*., 2017; Dhar *et al*., 2015a,b, 2016, 2017; Dias *et al*., 2013a,b; Kai *et al*., 2010, 2011; Li *et al*., 2013). The antibodies used in this study are listed in Table S1.

### 2.4 qRT‐PCR

The total RNA was isolated using the RNeasy Mini Kit (Qiagen, Hilden, Germany). qRT‐PCR was performed as described previously (Butt *et al*., 2017; Dhar *et al*., 2015a,b, 2016, 2017) on the LightCycler 480 II real‐time PCR instrument (Roche Diagnostics, Basel, Switzerland). All primers for qRT‐PCR are listed in Table S2.

### 2.5 ChIP‐Seq experiments and analysis

Two hundred milligrams of prostate tissues isolated from prostate‐specific *Pten* ^f/+^; *Pb‐Cre* ^+^ mouse, which had markedly increased MTA1 levels, was used for ChIP‐Seq assay. ChIP‐Seq was performed using MTA1 antibody (Bethyl Laboratories, Montgomery, TX, USA) at Active Motif (San Diego, CA, USA). Reads were aligned to the mouse genome, and peaks were identified (Dhar *et al*., 2016, 2017) using SICER algorithm. The ChIP‐Seq tracks presented here were generated using the UCSC Genome Browser.

### 2.6 Cell proliferation assay

PC3M‐Luc‐NS and PC3M‐Luc‐shMTA1 cells (2 × 10^3^) were seeded in a 35‐mm cell culture dish. The proliferation of these cells was determined by counting the cells every other day over a period of 10 days.

### 2.7 Colony formation assay

PC3M‐Luc‐NS and PC3M‐Luc‐shMTA1 cells (2 × 10^3^) were seeded in a 35‐mm cell culture dish for the 2‐week observation time. When colonies were freely visible (> 50 cells/colony), cells were fixed with formaldehyde and stained with 0.01% crystal violet solution. Colonies were analyzed by scanning and obtaining the image of each dish in the Bio‐Rad ChemiDoc Imager (Bio‐Rad Laboratories, Hercules, CA, USA). imagetool software (UTHSCSA, San Antonio, TX, USA) was used for counting the number of colonies in each dish.

### 2.8 Invasion assay

Invasion assay was performed using transwells having permeable support coated with Cultrex basement membrane extract (Corning Life Sciences, Corning, NY, USA) as described previously (Dhar *et al*., 2017). Briefly, 5 × 10^3^ serum‐starved PC3M‐Luc‐NS and PC3M‐Luc‐shMTA1 cells were seeded in serum‐free media in transwells and incubated for 24 h. After the incubation, cells in the upper membrane surface were scraped off with a cotton tip, whereas cells sticking to lower membrane surface were collected in cell dissociation solution containing calcein AM (Trevigen, Gaithersburg, MD, USA). This solution was then transferred to a black 96‐well plate, and fluorescence was read at 520‐/485‐nm excitation/emission. The relative fluorescence units were converted into cell number using the standard curve plotted with a serial dilution of cell numbers. Relative invasion was quantitated assuming 100% invasion for PC3M‐Luc‐NS control cells.

### 2.9 Wound healing assay

Motility changes in PC3M‐Luc MTA1 knockdown cells were determined by the wound healing assay as described previously (Dhar *et al*., 2017). Briefly, 90% confluent cells were starved in low serum media (0.1% serum), after which three separate wounds were scratched through the cells. The wound was imaged at 0, 12, 24, and 48 h using the Olympus CKX41 microscope with the Infinity Analyze software (Lumera Corporation, Bothell, WA, USA). Wound widths were calculated using the imagej software (NIH, Bethesda, MD). Migration index was quantitated assuming 100 for PC3M‐Luc‐NS control cells at 0 h.

### 2.10 Transgenic mouse model of prostate‐specific MTA1 overexpression

MTA1 transgenic founder mice were generated using CAG‐LoxP‐Stop‐LoxP(LSL)‐2HA‐MTA1‐T2A‐GFP‐pA construct to knock‐in the MTA1 transgene into the mouse ROSA26 (R26) locus (Applied StemCell, Inc., Milpitas, CA, USA). We crossbred these mice with Pb‐Cre4^+^ mice to obtain prostate‐specific overexpression of MTA1 (R26^MTA1/+^). Protein lysates from isolated prostate tissues were subjected to immunoblotting to confirm MTA1 overexpression.

### 2.11 Prostate cancer xenografts: subcutaneous and intracardiac

*Foxn1* ^nu/nu^ male mice were purchased from Envigo at 5–6 weeks of age. For s.c. xenografts, mice were randomly divided into two groups (*n* = 5 per group), and PC3M‐Luc‐NS and PC3M‐Luc shMTA1 cells (0.5 × 10^6^) in 100 μL of 50% Matrigel (Corning Life Sciences) were inoculated on the dorsal right flank of mice. Bioluminescent and caliper measurements were taken weekly as described previously (Dhar *et al*., 2015a,b; Dias *et al*., 2013a, b; Kai *et al*., 2011; Li *et al*., 2013). Calculation of tumor volume by digital vernier caliper measurements was made using formula as before (Dhar *et al*., 2015b; Kai *et al*., 2011). The mice were sacrificed at day 35. Tumors were excised from s.c. xenografts, total RNA and protein were isolated, and the remaining tissue was placed in 10% formalin for histological and immunohistochemical (IHC) analysis.

For intracardiac xenografts, mice were randomly divided into two groups (*n* = 5 per group), and PC3M‐Luc‐NS or PC3M‐Luc‐shMTA1 cells (0.1 × 10^6^) in 100 μL of 1× PBS were injected into the heart's left ventricle of anesthetized mice. Bioluminescent measurements were taken immediately and then weekly as described previously (Dhar *et al*., 2015a,b; Dias *et al*., 2013b; Kai *et al*., 2011; Li *et al*., 2013). The mice were sacrificed at day 34. The femur, tibia, maxilla, and mandible bones were harvested and placed in 10% formalin for bone histological analysis.

Housing and care of all animals were in accordance with the guidelines established by the University's Institutional Animal Care and Use Committee.

### 2.12 Histopathology and immunohistochemistry

Four‐micrometer‐thick formalin‐fixed paraffin‐embedded tumor and bone sections were processed and stained with H&E by Reveal Biosciences (San Diego, CA, USA). Immunohistochemical staining with Ki67, MTA1, CTSB, and E‐cad (antibodies are listed in Table S1) was performed as per the protocol described previously (Butt *et al*., 2017; Dhar *et al*., 2015a,b, 2016, 2017; Dias *et al*., 2013a; Kai *et al*., 2011; Li *et al*., 2013). The Vectastain ABC Elite Kit and ImmPACT DAB Kit (Vector Laboratories, Burlingame, CA, USA) were used to visualize staining. Images were recorded on an EVOS XL Core microscope (Thermo Fisher Scientific, Waltham, CA, USA). Ki67‐stained nuclei were quantitated using imagej software (NIH, Bethesda, MD).

### 2.13 Clinical samples

Fresh prostatectomy specimens were obtained immediately after surgery, and core biopsies were taken under sterile conditions using Coaxial Achieve Automatic Biopsy System (14G × 11 cm) as described previously (Dhar *et al*., 2017). The biopsy specimens were collected in vials and snap‐frozen. Two to three core biopsies were pooled together to extract protein for immunoblotting. All human studies were approved by University's Institutional Review Board. The experiments were undertaken with the understanding and written consent of each subject. The study methodologies confirmed to the standards set by the Declaration of Helsinki.

### 2.14 Statistical analysis

The differences between the groups were analyzed by the unpaired *t*‐test or one‐way ANOVA or two‐way ANOVA based on the experiment design. All tests were performed using graphpad prism 7 software (GraphPad Software, La Jolla, CA, USA). *P* < 0.05 was considered statistically significant. All data are cumulative of at least three independent experiments.

## 3 Results

### 3.1 MTA1 depletion impairs formation of bone metastasis, tumor growth, and progression in prostate cancer xenografts

To allow for noninvasive bioluminescence monitoring of bone metastases *in vivo*, we labeled the PC3M cells with luciferase (PC3M‐Luc) and further utilized these cells to establish cells expressing MTA1 (PC3M‐Luc‐NS) or silenced for MTA1 (PC3M‐Luc‐shMTA1). We chose the shMTA1#3 clone of PC3M cells silenced for MTA1 for our experiments based on its top knockdown efficiency among the tested shRNA clones and its *in vitro* luciferase activity equal to control PC3M‐Luc‐NS cells (Fig. S1).

**Figure 1:**
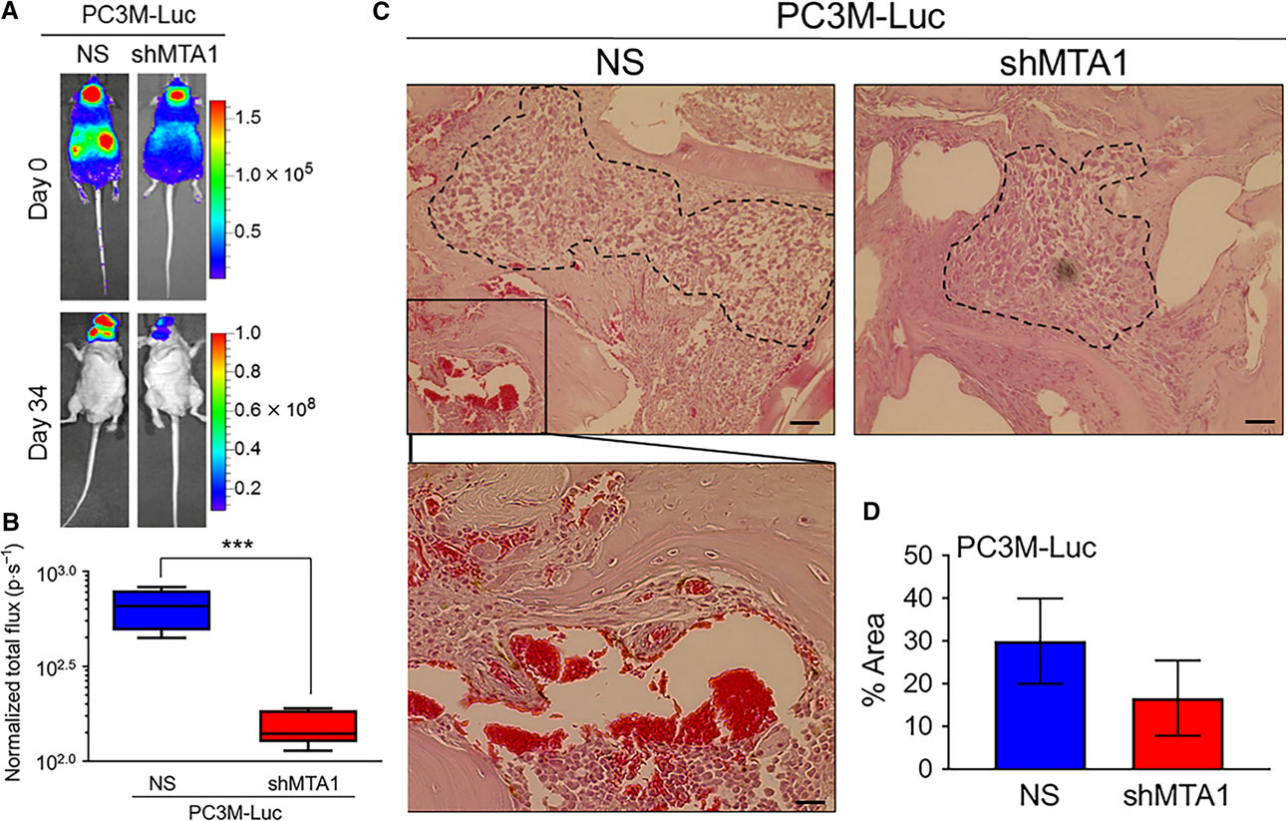
MTA1 knockdown suppresses formation of bone metastasis in experimental bone metastasis xenografts. (A) PC3M‐Luc‐NS and PC3M‐Luc‐shMTA1 cells were injected into the left ventricle of male athymic mice (*n* = 5) to colonize the bone. Bioluminescent images of representative tumor metastasis at day 0 (dorsal view) and day 34 (ventral view) are shown. (B) Quantitative analysis of bioluminescent signals of metastasis is shown in normalized total flux (photon per sec). ***P < 0.001 (unpaired *t*‐test). (C) Representative histology for bone metastatic lesions from two groups of mice is shown. Scale bars, 50 μm. Scale bar (magnified image), 10 μm. (D) Quantitation of the metastatic carcinoma area. Data represent the mean ± SEM of metastatic area from three mice in each group.

PC3M cells were able to develop visceral metastasis in orthotopic prostate cancer xenografts, but no metastasis to the bone was detected (K. Li, S. Dias, S. Dhar, M. Ivanovic & A. S. Levenson, unpublished data). Therefore, to delineate the role of MTA1 in bone metastasis, we utilized the experimental bone metastasis model with injections of cells into the left cardiac ventricle of athymic mice (Chong Seow Khoon, 2015; Holzapfel *et al*., 2014; Miftakhova *et al*., 2016; Rosol *et al*., 2004). Metastases were monitored by weekly bioluminescence imaging until day 34, when mice were sacrificed. We found bioluminescent signal localizing only to the jawbones (maxilla and mandible). Representative image (Fig. 1A) and quantitation of normalized total flux (Fig. 1B) showed statistically significant reduction in jawbone‐localized bioluminescent signal in PC3M‐Luc shMTA1 compared to NS control xenografts, suggesting that MTA1 depletion significantly compromised the colonization of PC3M prostate cancer cells to the bone. H&E staining of jawbones showed that prostate cancer cells infiltrated the bone and formed metastatic tumor foci (Fig. 1C). Notably, the NS control metastatic bone lesions exhibited areas of intense bony remodeling (Fig. 1C, magnified image). Further, we found that tumor foci in NS control bone metastatic lesions occupied a greater percentage of the histological section area compared to shMTA1 lesions (Fig. 1C, D), although results were statistically not significant. These data suggest that cells lacking MTA1 had impaired ability to colonize and form bone metastasis compared to cells expressing high levels of MTA1.

**Figure 2:**
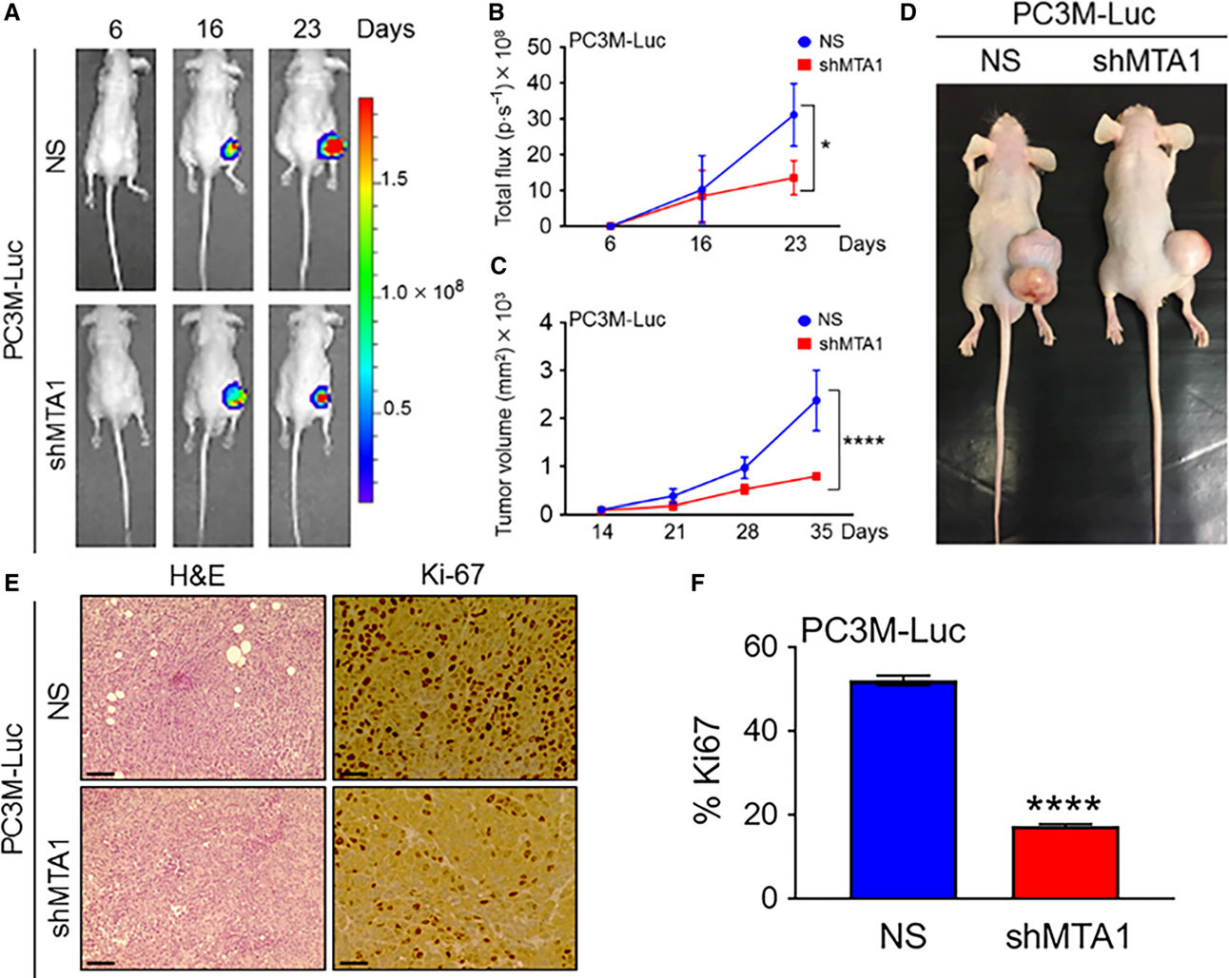
MTA1 knockdown effectively diminishes tumor growth and progression in s.c. tumor xenografts. (A) PC3M‐Luc‐NS and PC3M‐Luc‐shMTA1 cells were injected s.c. into the male athymic mice (*n* = 5). Dorsal views of bioluminescent (BL) images of representative tumor xenografts at days 6, 16, and 23 are shown. (B) Quantitative analysis of tumor light emission from day 6 after cancer cell injections until day 23 is shown in Total Flux (photon per sec). *P < 0.05 (two‐way ANOVA). (C) Tumor growth was monitored using a digital caliper once a week starting at day 14. ****P < 0.0001 (two‐way ANOVA). (D) Representative mouse tumor xenografts showing MTA1 depletion effect on tumor growth. (E) H&E‐stained sections (scale bars, 100 μm) and IHC analyses (scale bars, 50 μm) for proliferative activity (Ki67) in tumor tissues from PC3M‐Luc‐NS and PC3M‐Luc‐shMTA1 xenografts. (F) Quantitation of proliferative index in five randomly selected fields for each tissue. Data represent the mean ± SEM of five fields from five mice. ****P < 0.0001 (unpaired *t*‐test).

We have previously reported on MTA1 knockdown inhibiting the tumor growth and progression of the LNCaP and DU145 s.c. prostate cancer xenografts (Kai *et al*., 2011). To determine the contribution of MTA1 to the tumor growth and progression of the PC3M aggressive prostate cancer cells *in vivo*, PC3M‐Luc‐NS and PC3M‐Luc‐shMTA1 cells were injected s.c. into the right flank of athymic male nude mice. Both bioluminescent imaging and caliper measurements were taken to monitor tumor growth and progression weekly. Bioluminescence measurement of tumors showed drastically reduced tumor growth in PC3M‐Luc‐shMTA1 compared to PC3M‐Luc‐NS control xenografts (Fig. 2A), as evident by significant differences in photon emission by day 23 (Fig. 2B). As after a certain point of time s.c. tumors give nonconsistent bioluminescent measurements due to limitations of photon emission detection in large tumors with necrotic areas (Dhar *et al*., 2015a,b; Dias *et al*., 2013b; Kim *et al*., 2010; Rehemtulla *et al*., 2000), caliper‐based measurements, which were feasible after day 14, became more reliable. Figure 2C, D shows highly significant differences in tumor volumes between control and shMTA1 xenografts by day 35, when mice were sacrificed. Immunohistochemical analysis of tumors removed from xenografts revealed that the tumors formed from the PC3M‐Luc‐shMTA1 cells had lower proliferative index compared to PC3M‐Luc‐NS controls, as indicated by decreased number of Ki67‐positive cells in PC3M‐Luc‐shMTA1 tumors (Fig. 2E, F). These data indicate that silencing of MTA1 impairs tumor growth and progression in PC3M prostate cancer xenografts.

The changes in body weight of mice from both the NS control and shMTA1 groups were not significantly different during the intracardiac and s.c. xenograft experiments (data not shown).

### 3.2 MTA1 depletion suppresses the metastatic potential of human prostate cancer cells

**Figure 3:**
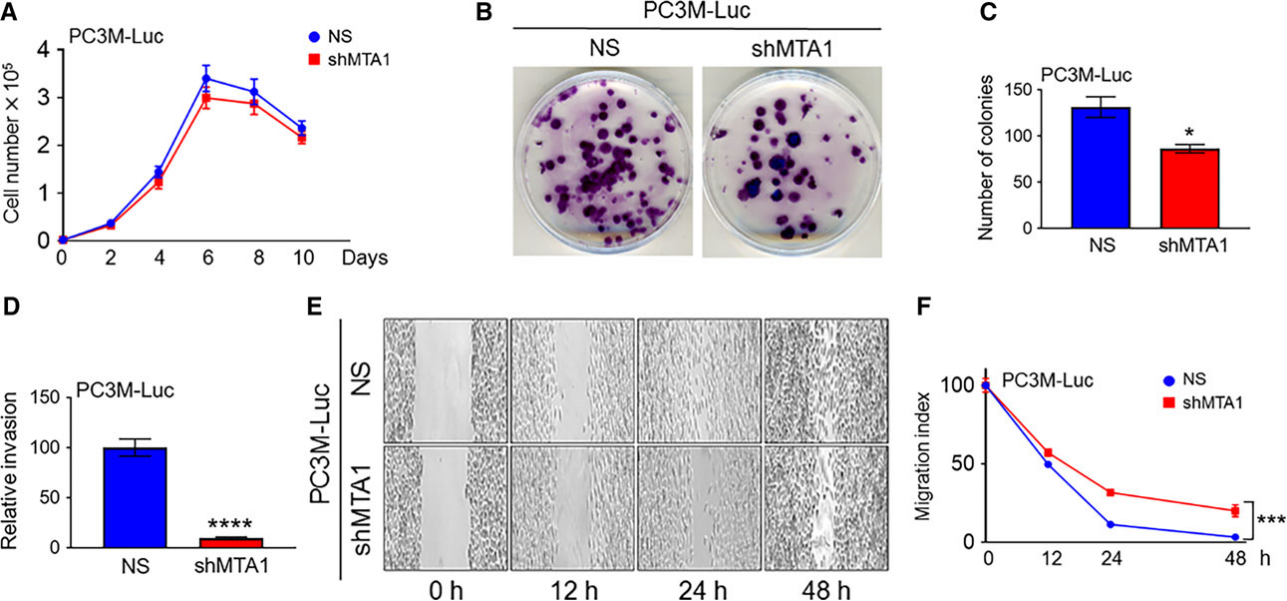
MTA1 knockdown reduces metastatic potential of PC3M‐Luc prostate cancer cells. (A) Proliferation assay of PC3M‐Luc‐NS and PC3M‐Luc‐shMTA1 cells. (B) Representative images of colony formation ability of PC3M‐Luc‐NS and PC3M‐Luc‐shMTA1 cells and (C) quantitation of colonies are shown. Data represent the mean ± SEM of three independent experiments. *P < 0.05 (unpaired *t*‐test). (D) Invasion assay in PC3M‐Luc‐NS and PC3M‐Luc‐shMTA1 cells. Data represent the mean ± SEM of three independent experiments. ****P < 0.0001 (unpaired *t*‐test). (E) Representative images of wound healing assay to compare migration ability of PC3M‐Luc‐NS and PC3M‐Luc‐shMTA1 cells and (F) quantitation of wound widths as migration index are shown. Data represent the mean ± SEM of six separate wounds and three independent experiments. ***P < 0.001 (two‐way ANOVA).

To determine the cellular mechanisms through which depletion of MTA1 impedes formation of bone metastasis, we performed cell proliferation, colony formation, invasion, and wound healing assay *in vitro* using PC3M‐Luc‐NS and PC3M‐Luc‐shMTA1 cells. Although the PC3M‐Luc cells silenced for MTA1 (shMTA1) proliferated at a rate slightly slower than the control cells (NS), the differences in proliferation were not statistically significant (Fig. 3A). Notably, however, MTA1 knockdown inhibited the colony‐forming ability of PC3M‐Luc cells, as is evident by a significant decrease in the number of colonies formed by shMTA1 compared to the NS control cells (Fig. 3B, C). Further, MTA1 silencing in PC3M cells significantly altered their invasive and wound healing ability, an *in vitro* measure of cell metastatic potential. PC3M‐Luc cells silenced for MTA1 (shMTA1) exhibited about 90% reduction in invasiveness (Fig. 3D) and about 20% decrease in wound closure (Fig. 3E, F) as compared to NS controls. These data reveal that MTA1 silencing decreases metastatic potential of PC3M‐Luc cells *in vitro*, which in turn may be responsible, at least in part, for the reduced formation of bone metastases in intracardiac xenografts generated using cells lacking MTA1.

### 3.3 MTA1 knockdown reduces cathepsin B in prostate cancer cells and tumor xenografts

**Figure 4:**
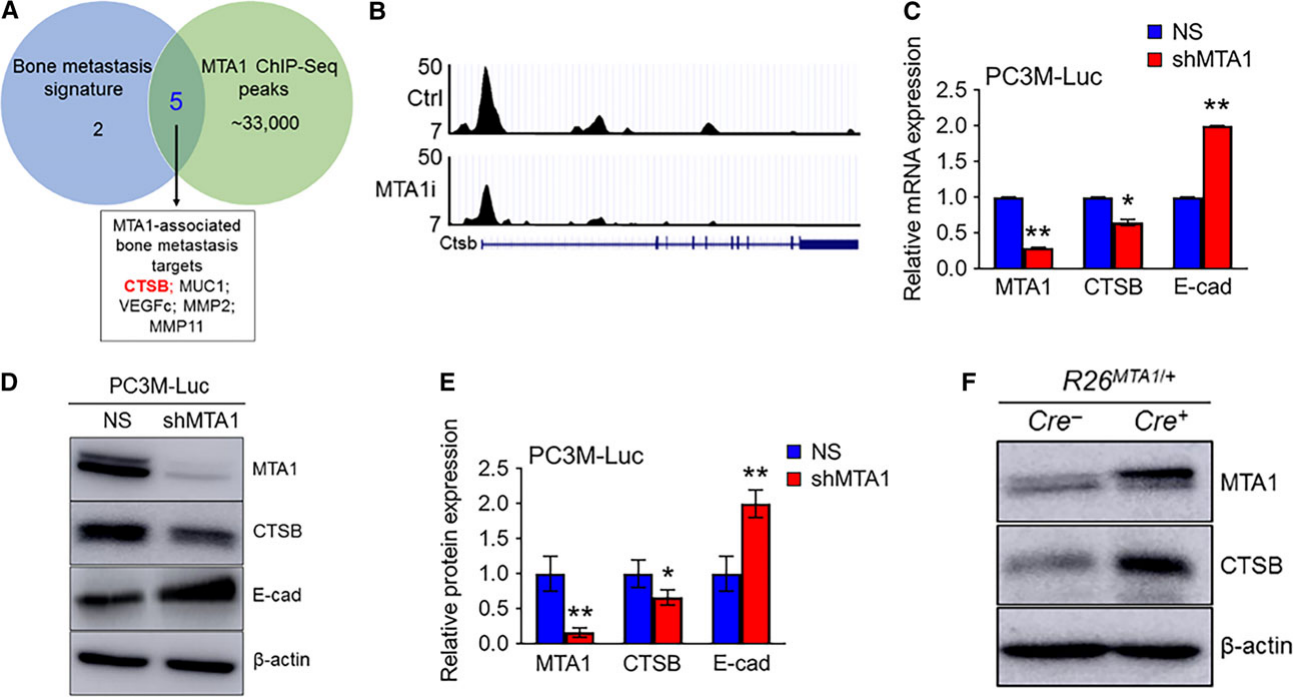
MTA1 knockdown reduces cathepsin B mRNA and protein levels in PC3M‐Luc prostate cancer cells. (A) Venn diagram depicting a number of genes selected as bone metastatic signature (Kai *et al*., 2011), number of peaks identified by MTA1 ChIP‐Seq (Dhar *et al*., 2016), and overlapping MTA1‐associated bone metastasis targets of interest. (B) Comparative analysis of ChIP‐Seq data on MTA1 binding in the prostate tissue of *Pten* ^+/−^ mice expressing high levels of MTA1 (Ctrl, upper panel) and reduced levels of MTA1 (MTA1i, lower panel). Representative MTA1 ChIP‐Seq track for CTSB gene loci at 10‐kb resolution is shown. (C) qRT‐PCR of MTA1, CTSB, and E‐Cad mRNA levels in PC3M‐Luc‐NS and PC3M‐Luc‐shMTA1 cells. (D) Immunoblots of MTA1, CTSB, and E‐cad in PC3M‐Luc‐NS and PC3M‐Luc‐shMTA1 cells. β‐Actin was used as a loading control. (E) Quantitation of immunoblot signals. Data represent the mean ± SEM of three independent experiments. *P < 0.05; **P < 0.01 (two‐way ANOVA). (F) Immunoblots of MTA1 and CTSB in prostate tissues from 13‐week‐old *Pb‐Cre* ^+^; *R26* ^*MTA*^ ^*1*/+^ mice and Cre‐negative normal prostate control.

In our previous studies, we have identified key genes involved in the ‘vicious cycle’ interaction between prostate cancer cells and bone cells *in vitro* (Kai *et al*., 2011). This ‘bone metastasis signature’ comprised eight genes including vascular endothelial growth factor‐c; matrix metalloproteinases MMP2, MMP10, and MMP11; cathepsins B and K (CTSB and CTSK); mucin 1 (MUC1); and MTA1. In an attempt to dissect molecular mechanisms through which MTA1 promotes bone metastasis, we performed integrative analysis of our bone metastasis signature data along with our MTA1 ChIP‐Seq data and found CTSB as a strong potential candidate responsible for MTA1‐driven invasiveness (Fig. 4A). Furthermore, our MTA1 ChIP‐Seq data demonstrated that pharmacological inhibition of MTA1 (MTA1i) resulted in reduced CTSB promoter occupancy by MTA1, suggesting a possible transcriptional regulation of CTSB by MTA1 (Fig. 4B).

To further characterize the MTA1‐CTSB interrelationship, we examined the expression of cathepsin B in PC3M‐Luc control (NS) and MTA1 knockdown (shMTA1) cells. As shown in Fig. 4C–E, both mRNA and protein levels of CTSB were significantly decreased in PC3M‐Luc cells silenced for MTA1 (shMTA1) compared to control (NS) cells. In agreement with our previous reported finding of MTA1 and E‐cadherin (E‐cad) inverse relationship in prostate cancer (Dhar *et al*., 2016, 2017), shMTA1 cells showed an expected increase in E‐cad levels compared to NS control cells (Fig. 4C–E). Additionally, LNCaP cells silenced for MTA1 also exhibited reduced levels of CTSB (Fig. S2). Conversely, prostate tissues from our prostate‐specific MTA1‐overexpressing mice (R26^MTA1/+^; Cre4^+^), which express significantly reduced levels of E‐cad (Dhar *et al*., 2017), also exhibited significantly increased CTSB levels (Fig. 4F), thereby further strengthening our observation of a MTA1‐CTSB positive relationship.

**Figure 5:**
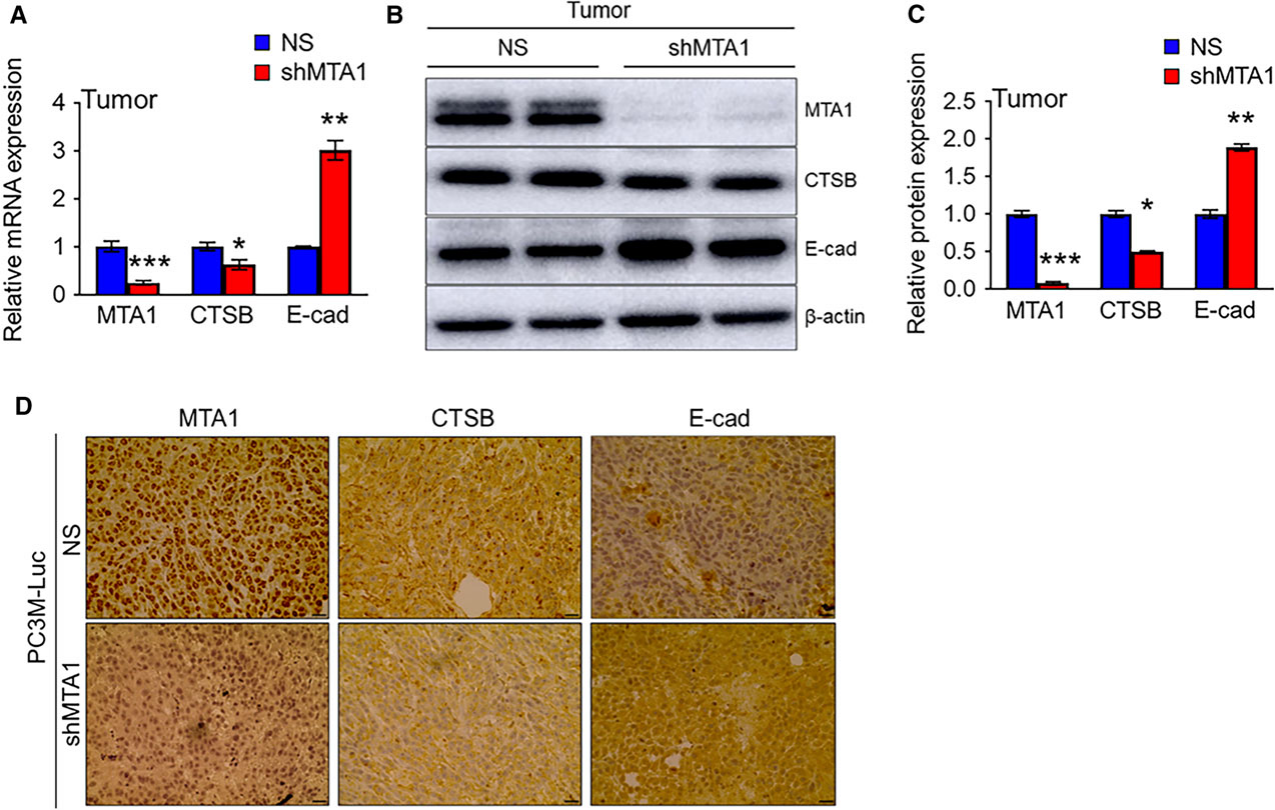
MTA1 knockdown reduces cathepsin B mRNA and protein levels in s.c. tumor tissues. (A) qRT‐PCR of MTA1, CTSB, and E‐cad mRNA levels in the tumor tissues from s.c. xenografts. (B) Immunoblots of MTA1, CTSB, and E‐cad in the tumor tissues from s.c. xenografts. β‐Actin was used as a loading control. (C) Quantitation of immunoblot. Data represent the mean ± SEM of three independent experiments from *n* = 3 for qRT‐PCR and *n* = 2 for immunoblot analysis. *P < 0.05; **P < 0.01; ***P < 0.001 (two‐way ANOVA). (D) Comparison of nuclear MTA1 and cytoplasmic CTSB and membrane‐bound E‐cad IHC staining in the tumor tissues from PC3M‐Luc‐NS and PC3M‐Luc‐shMTA1 xenografts. Scale bars, 10 μm.

Of note, consistent with our *in vitro* data, qRT‐PCR and immunoblot analysis revealed that the mRNA and protein expressions of CTSB were significantly reduced while E‐cad mRNA and protein levels were considerably increased in the tumor tissues derived from MTA1 knockdown (shMTA1) cells compared to NS controls (Fig. 5A–C). Immunohistochemical analysis of these tumors showed an expected decreased MTA1 nuclear staining in MTA1 knockdown (shMTA1) compared to intense staining in control (NS) tissues. Accordingly, we detected reduced CTSB cytoplasmic protein levels and elevated E‐cad membrane‐bound staining in MTA1 knockdown (shMTA1) compared to MTA1‐expressing (NS) xenografts (Fig. 5D). These data suggest that positive regulation of CTSB expression by MTA1 in prostate tumor cells may be, at least in part, responsible for promotion of prostate tumor growth and bone metastasis formation.

### 3.4 MTA1 and CTSB expressions show a strong positive correlation in prostate cancer clinical samples

**Figure 6:**
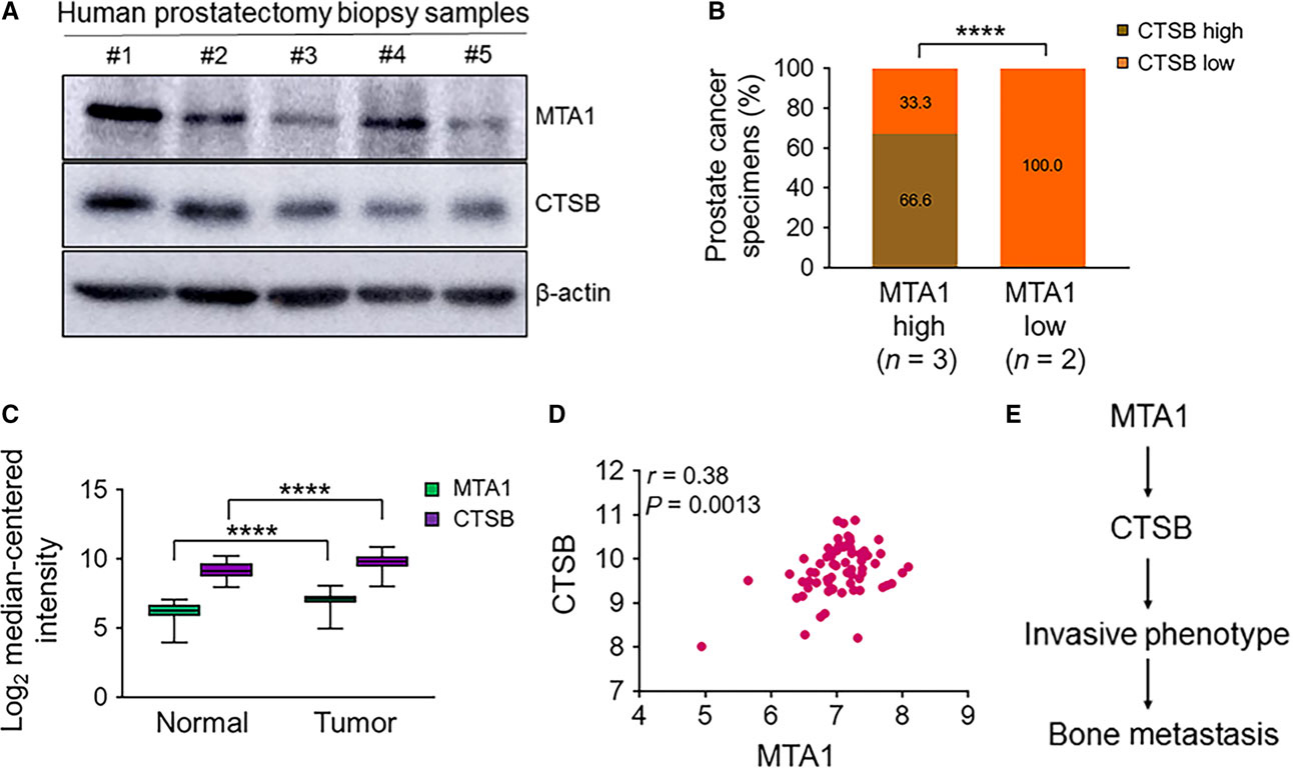
MTA1/CTSB axis in human prostate cancer. (A) Immunoblot analysis for MTA1 and CTSB protein levels in five representative patients. (B) Distribution of MTA1 and CTSB expression in the cohort of patients with prostate cancer analyzed in (A). ****P < 0.0001 (chi square test). (C) Meta‐analysis showing MTA1 and CTSB mRNA overexpression in prostate tumor samples compared to normal tissues from expression array (Wallace *et al*., 2008) using the Oncomine database. ****P < 0.0001 (two‐way ANOVA). (D) Meta‐analysis of MTA1 and CTSB mRNA expression showing a strong positive correlation in prostate tumor samples from expression array (Wallace *et al*., 2008) using the Oncomine database. (E) Schematic of the proposed MTA1‐mediated cathepsin B involvement in prostate cancer metastasis to the bone.

To explore the clinical significance of the MTA1 and CTSB interrelationship in prostate cancer, we examined the expression of MTA1 and CTSB in a limited cohort of five prostate adenocarcinoma biopsies obtained previously (Dhar *et al*., 2017). We found a strong positive correlation between MTA1 and CTSB protein expressions: two of three biopsies with high levels of MTA1 had high CTSB expression (Fig. 6A, B). To strengthen our findings from smaller sample numbers, we analyzed the publicly available prostate cancer patient dataset (Wallace *et al*., 2008) from Oncomine database and found significantly higher levels of both MTA1 and CTSB in tumor samples compared to normal prostate tissues (Fig. 6C). Of note, MTA1 and CTSB exhibited a strong positive correlation from 69 prostate cancer patient samples (Wallace *et al*., 2008) (Fig. 6D, *r* = 0.38). In addition, copy number analysis data of the prostate cancer patient samples from independent studies showed an amplification of MTA1 and CTSB (Fig. S3). These data indicate that CTSB is overexpressed in prostate tumors and that this may depend, at least in part, on the MTA1‐associated transcriptional induction of CTSB.

## 4 Discussion

Various mechanisms are associated with metastatic process of primary tumor including interactions of tumor cells with reactive stroma and extracellular matrix and altered cell attachment. Although successful tumor invasion requires degradation of extracellular matrices and EMT by effector molecules such as proteases and cadherins, the key role of upstream master regulators of these effector molecules in the promotion of invasiveness leading to metastasis cannot be undermined.

We have previously demonstrated the key upstream regulatory role of MTA1 in processes involved in development and progression of prostate cancer (Dhar *et al*., 2016). High levels of MTA1 were detected in bone metastatic lesions of human prostate cancer patients (Dias *et al*., 2013a; Kai *et al*., 2011). At the same time, CTSB has been shown to play an important role in invasion and metastasis of prostate cancer (Miyake *et al*., 2004). Increased CTSB expression and activity were observed in bone tissues colonized by human prostate carcinoma cells (Podgorski *et al*., 2005). It was suggested that metabolically rich bone microenvironment is responsible for secretion of active CTSB from prostate cancer cells interacting with the bone (Podgorski *et al*., 2005). Our unpublished data also show induction of CTSB expression in prostate cancer cells, when cocultured with bone cells than when cultured alone. While we fully agree that complex tumor–host interactions can modulate the expression of proteins involved in the ‘vicious cycle’ of bone metastasis, we believe that regulating CTSB expression and activity in the primary tumor might be a way of preventing the development of site metastasis, including the bone.

In this study, we have demonstrated that MTA1 promotes prostate tumor growth and formation of bone metastasis, at least in part, by positively regulating CTSB expression in the primary tumor cells. Opposed to effects on CTSB and as expected based on our previous observations (Dhar *et al*., 2016, 2017), MTA1 negatively regulates E‐cad expression in primary tumor, a hallmark of EMT associated with metastasis (Figiel *et al*., 2017), indicating the impact of MTA1 in the process of EMT and cancer cell ability to migrate to distant sites.

Marked increase in MTA1 levels, which is an indicator of advanced castrate‐resistant prostate tumors, might be an important factor in endorsing CTSB‐mediated degradation of basement membrane and in the ability of primary prostate tumor cells to undergo E‐cad‐mediated EMT. We believe that a functional interaction between MTA1 and these two downstream pathways is critical for the acquisition of an invasive phenotype by tumor cells, offering new therapeutic approaches for targeting MTA1‐positive high‐risk tumors to prevent metastases. Of note, the fact that our intracardiac shMTA1 xenografts exhibited reduced formation of bone metastasis suggests that it is possible to prevent homing of primary prostate tumor cells to the bone microenvironment by targeting MTA1.

Thus, MTA1 is an attractive target for the treatment of prostate cancer and prevention of bone metastasis. Our group has intensively published on pharmacological inhibition of MTA1 and its associated signaling by natural dietary compounds such as resveratrol and pterostilbene as well as PI3K and histone deacetylase inhibitors resulting in targeted chemoprotective and anticancer effects in various prostate cancer model systems including xenografts and transgenic mice (Butt *et al*., 2017; Dhar *et al*., 2015a,b, 2016, 2017; Kai *et al*., 2010; Kumar *et al*., 2015; Levenson *et al*., 2014; Li *et al*., 2013). Particularly, we have demonstrated that inhibition of MTA1 by pterostilbene restored E‐cad expression in the prostate tissues of the conditional *Pten* null mice (Dhar *et al*., 2016). We have also demonstrated the superior MTA1‐targeted anticancer efficacy of pterostilbene/SAHA combination strategy in prostate cancer (Butt *et al*., 2017).

## 5 Conclusions

In conclusion, our study provides mechanistic and preclinical insight into the critical role of MTA1 in tumor progression and formation of bone metastasis in prostate cancer, at least in part, through the CTSB‐dependent promotion of metastatic pathways (Fig. 6E). Our results offer the MTA1/CTSB axis as a promising target for novel molecular therapies in prostate cancer bone metastasis.

## Author contributions

ASL conceived the project. AK, SD, and ASL designed the research. AK, SD, GC, NAB, and JMS performed experiments. AK, SD, JMS, CRG, and ASL analyzed the data. AK and ASL wrote the manuscript. All authors critically revised and approved the manuscript.

## Supporting information


**Fig. S1.** Establishment of PC3M‐Luc MTA1 knockdown cells.
**Fig. S2.** MTA1 knockdown reduces cathepsin B protein levels.
**Fig. S3.** CTSB expression correlates with MTA1 expression in human prostate cancer.
**Table S1.** Antibodies for Immunoblots and IHC used in this study.
**Table S2.** Primers for qRT‐PCR used in this study.
