# Supplementary material for: MTA1 drives malignant progression and bone metastasis in prostate cancer
Source: Mol Oncol. 2018 Aug 14;12(9):1596–607. doi: 10.1002/1878-0261.12360 (PMC6120234; doi:10.1002/1878-0261.12360)
Supplement: Supplementary file 1 — Fig. S1. Establishment of PC3M‐Luc MTA1 knockdown cells. Fig. S2. MTA1 knockdown reduces cathepsin B protein levels. Fig. S3. CTSB expression correlates with MTA1 expression in human prostate cancer. Table S1. Antibodies for Immunoblots and IHC used in this study. Table S2. Primers for qRT‐PCR used in this study. [file MOL2-12-1596-s001.pdf]

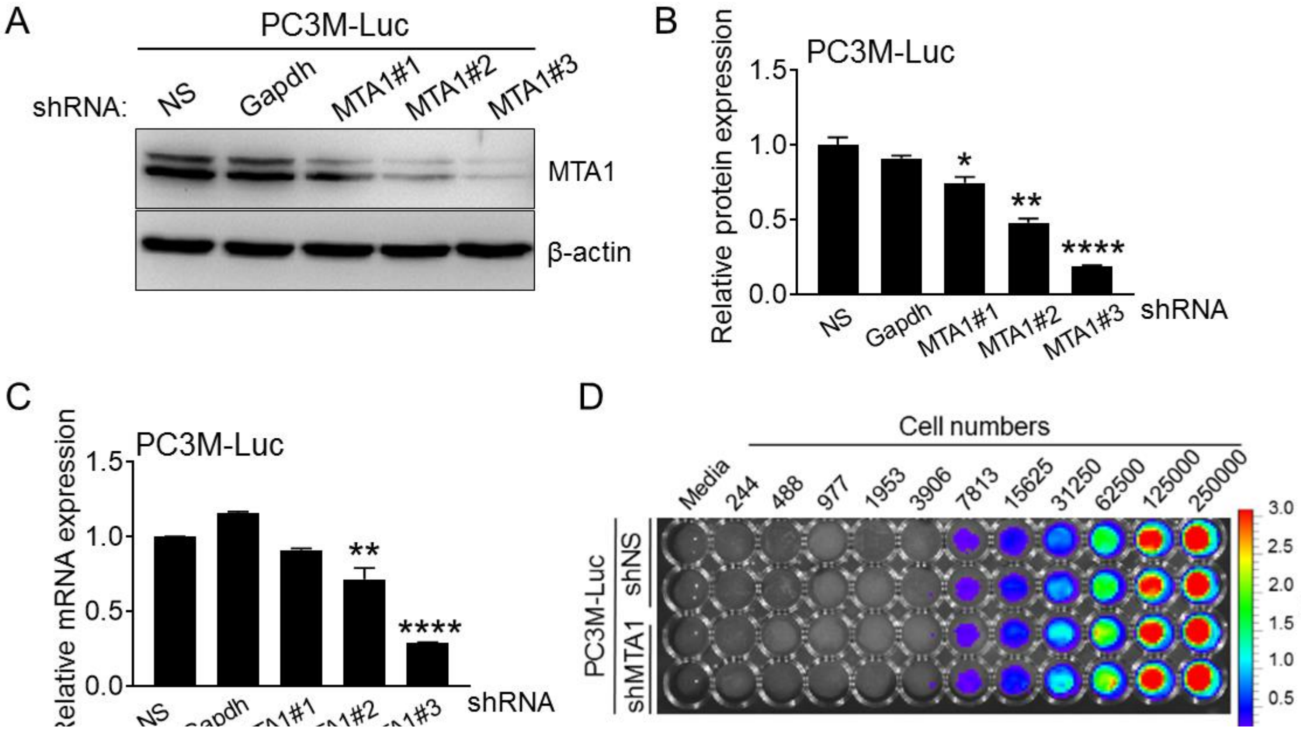

Fig. S1. Establishment of PC3M-Luc MTA1 knockdown cells. (A) Immunoblot of MTA1 protein expression in cells transfected with NS (Non-Silencing negative control), Gapdh shRNA (specificity control) and three MTA1 shRNAs (shMTA1#1, shMTA1#2 and shMTA1 #3). β-actin was used as a loading control. (B) Quantitation of immunoblot signals. (C) Expression of MTA1 mRNA levels analyzed by qRT-PCR. Levels were normalized relative to NS Ctrl values setting at 1. *$p < 0.05$; **$p < 0.01$; ****$p < 0.0001$ (one-way ANOVA). (D) *In vitro* validation of equal luciferase (Luc) expression in PC3M-Luc-NS and PC3M-Luc-shMTA1#3 cells.

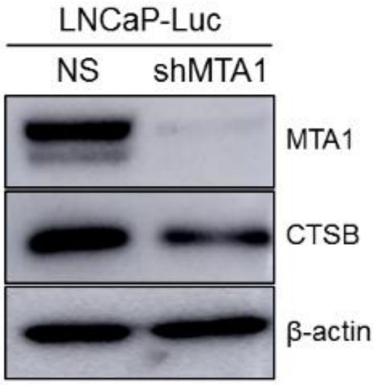

Fig. S2. MTA1 knockdown reduces cathepsin B protein levels. Immunoblots of MTA1 and CTSB in LNCaP-Luc-NS and LNCaP-Luc-shMTA1 cells. β-actin was used as a loading control.

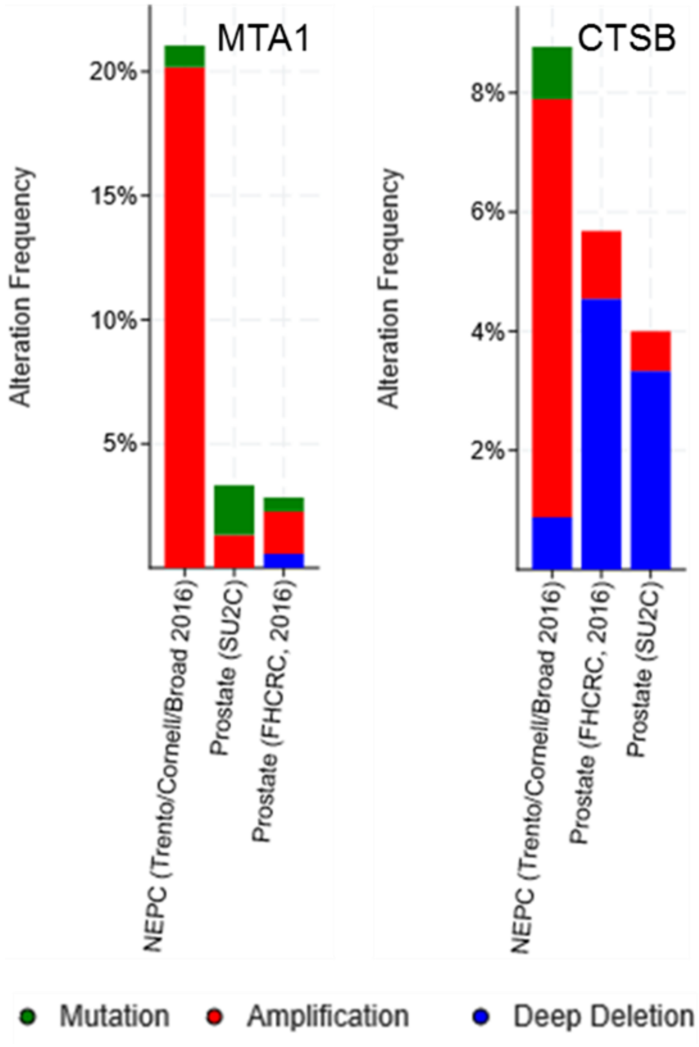

Fig. S3. CTSB expression correlates with MTA1 expression in human prostate cancer. Copy number analysis (CNA) and mutation analysis for MTA1 and CTSB in publicly available prostate cancer patient datasets using cBioPortal for Cancer Genomics.

Table S1. Antibodies for Immunoblots and IHC used in this study.

**Antibody**	**Method**	**Dilution**	**Source**	**Catalog#**
MTA1	Immunoblot	1:1000	Cell Signaling Technologies	5647
	IHC	1:50		
Cathepsin B	Immunoblot	1:1000	Cell Signaling Technologies	31718
	IHC	1:500		
E-cadherin	Immunoblot	1:1000	Cell Signaling Technologies	3195
	IHC	1:400		
Ki67	IHC	1: 100	Abcam	ab16667
β-actin	immunoblot	1:2500	Santa Cruz Biotechnology	sc-69879

Table S2. Primers for qRT-PCR used in this study.

**Primers**	**Sequence**
MTA1 forward	5'- AGC TAC GAG CAG CAC AAC GGG GT - 3'
MTA1 reverse	5'- CAC GCT TGG TTT CCG AGG AT - 3'
CTSB forward	5'- CAC TGA CTG GGG TGA CAA TG - 3'
CTSB reverse	5'- GCC ACC ACT TCT GAT TCG AT - 3'
E-cad forward	5' - GAA CGC ATT GCC ACA TAC AC - 3'
E-cad reverse	5' - ATT CGG GCT TGT TGT CAT TC - 3'
β-actin forward	5'- CGT GGG CCG CCC TAG GCA CCA - 3'
β-actin reverse	5'- TTG GCT TAG GGT TCA GGG GGG - 3'
